# Two mitochondrial genomes of freshwater gudgeons in the genus *Gobio* (Cypriniformes: Gobionidae)

**DOI:** 10.1080/23802359.2020.1797569

**Published:** 2020-07-30

**Authors:** Tianyu Yi, Cuizhang Fu

**Affiliations:** Ministry of Education Key Laboratory for Biodiversity Science and Ecological Engineering, Coastal Ecosystems Research Station of the Yangtze River Estuary, Institute of Biodiversity Science and Institute of Eco-Chongming, School of Life Sciences, Fudan University, Shanghai, China

**Keywords:** *Gobio*, *Romanogibio*, Cypriniformes, Gobionidae, East Asia

## Abstract

Two complete mitochondrial genomes of *Gobio acutipinnatus* and *G. microcephalus* are assembled, and they have the same length with slightly high A + T contents (54.88% and 56.57%) in base compositions. Results in the reconstructed phylogeny show that the genus *Gobio* is a monophyletic group, and it is a sister taxon of the genus *Romanogibio*.

Freshwater gudgeons in the genus *Gobio* (Cypriniformes: Gobionidae) include about 46 valid species (Fricke et al. 2020), and they are distributed in Europe and Asia (Yue 1998; Kottelat and Freyhof 2007). In this study, we assemble complete mitochondrial genomes of *Gobio acutipinnatus* and *Gobio macrocephalus* through Sanger sequencing. The two new mitochondrial genomes would be useful in species delimitation and phylogenetic reconstruction of the genus *Gobio*.

The specimen of *G. acutipinnatus* (voucher FDZM-GABu20150427-01) is collected from the Burqin County, Xinjiang Uygur Autonomous Region, China (47.71°N, 86.84°E), and *G. macrocephalus* (FDZM-GMWQ20170804-01) from the Wangqing County, Jilin Province, China (43.32°N, 129.78°E).

Two new mitochondrial genomes (MT632635 and MT632636 in the GenBank) have the same length (16, 609 bp), and display slightly high A + T contents (54.88% and 56.57%) in base compositions. There are two kinds of start codons (ATG and GTG) and four types of stop codons (TAG, TAA, TA– and T––) used in the 13 protein-coding genes. Gene overlaps are revealed among 6 pairs of adjoining genes with variable lengths of 1–7 bp, as well as gene intervals among 13 pairs of adjacent genes with different size of 1–31 bp. All of gene compositions, the order of genes, codon uses, overlaps and intervals among genes show similar patterns as published mitochondrial genomes of their close relatives in the family Gobionidae (Li et al. 2018; Chen and Fu 2019; Ge et al. 2020).

A phylogeny is reconstructed by combining with other 19 mitochondrial genomes of the family Gobionidae from the GenBank, using a maximum likelihood analysis in the IQ-TREE 1.6.2 (Nguyen et al. 2015). The results in our phylogeny show that the genus *Gobio* is a monophyletic group, and it forms a sister taxon relationship with the genus *Romanogibio* (Figure 1).

**Figure 1.**
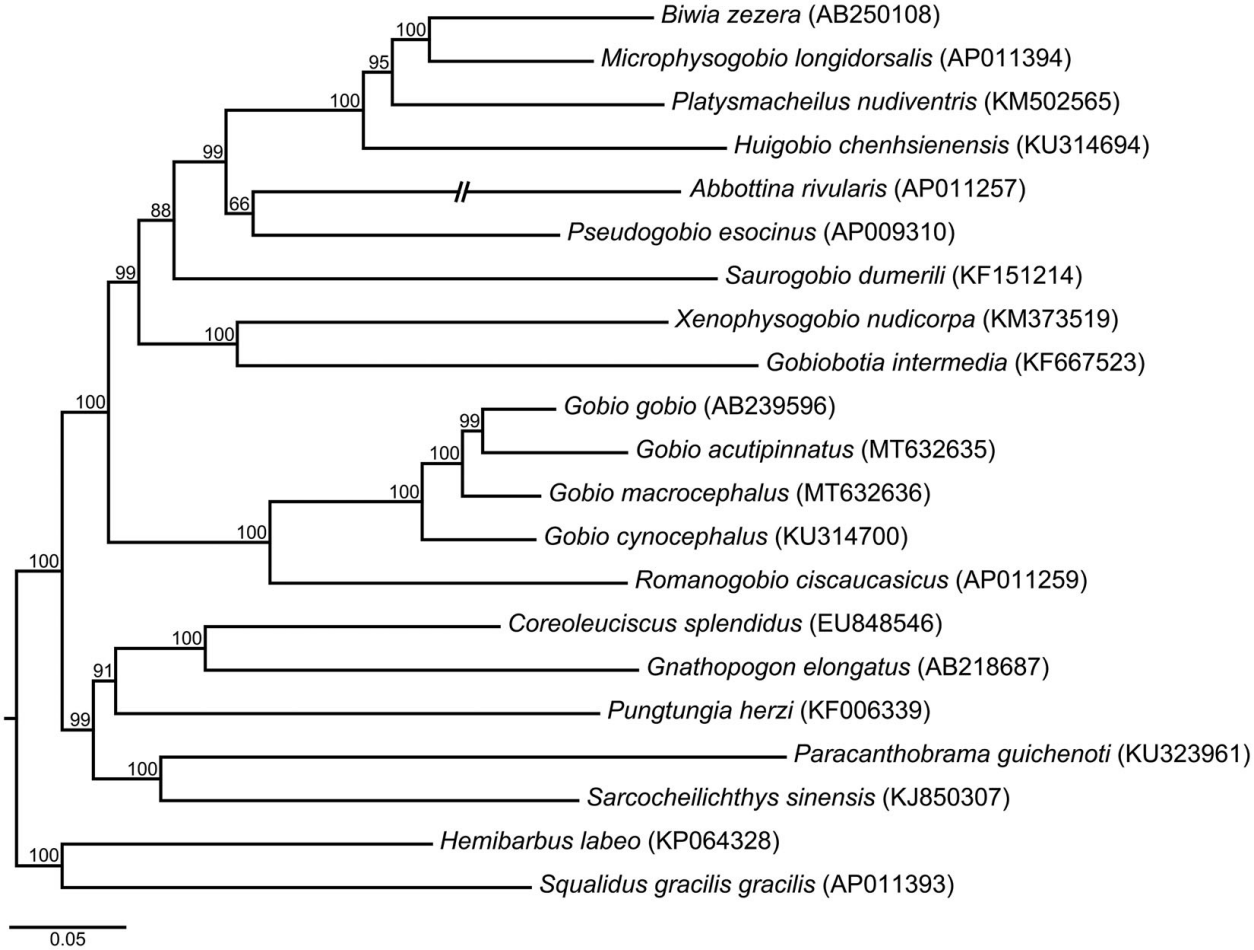
Phylogenetic relationships between *Gobio* fishes and their relatives using 21 mitochondrial genomes based on a maximum likelihood analysis. Shown are bootstrap confidences above branches, and GenBank numbers in parentheses.

## Data Availability

New mitochondrial genomes in this study could been obtained in the GenBank with accession numbers as follows: MT632635 (https://www.ncbi.nlm.nih.gov/nuccore/MT632635) MT632636 (https://www.ncbi.nlm.nih.gov/nuccore/MT632636)
